# Differential regulation of apoptosis-related genes during long-term culture and differentiation of canine adipose-derived stem cells - a functional bioinformatical analysis

**DOI:** 10.3389/fgene.2024.1515778

**Published:** 2025-01-06

**Authors:** Maurycy Jankowski, Katarzyna Stefańska, Michał Suchodolski, Claudia Dompe, Grzegorz Wąsiatycz, Bartosz Kempisty, Michał Nowicki, Magdalena Roszak

**Affiliations:** ^1^ Department of Computer Science and Statistics, Poznan University of Medical Sciences, Poznan, Poland; ^2^ Deparment of Histology and Embryology, Poznan University of Medical Sciences, Poznan, Poland; ^3^ Greater Poland Center of Digital Medicine, Poznan University of Medical Sciences, Poznan, Poland; ^4^ Department of Immunology, Poznan University of Medical Sciences, Poznan, Poland; ^5^ Doctoral School, Poznan University of Medical Sciences, Poznan, Poland; ^6^ Department of Human Morphology and Embryology, Division of Anatomy, Faculty of Medicine, Wrocław Medical University, Wrocław, Poland; ^7^ Institute of Veterinary Medicine, Nicolaus Copernicus University, Torun, Poland; ^8^ Physiology Graduate Faculty, North Carolina State University, Raleigh, NC, United States; ^9^ Center of Assisted Reproduction, Department of Obstetrics and Gynecology, University Hospital and Masaryk University, Brno, Czechia

**Keywords:** adipose tissue, stem cells, transcriptomics, gene expression, gene ontology, apoptosis

## Abstract

**Introduction:**

Stem cells derived from adipose tissue are gaining popularity in the field of regenerative medicine due to their adaptability and clinical potential. Their rapid growth, ability to differentiate, and easy extraction with minimal complications make adipose-derived stem cells (ADSCs) a promising option for many treatments, particularly those targeting bone-related diseases. This study analyzed gene expression in canine ADSCs subjected to long-term culture and osteogenic differentiation.

**Methods:**

ADSCs were isolated from discarded surgical waste and cultured for 14 days with and without differentiation media to assess osteogenic changes. RNA sequencing (RNA-seq) and bioinformatical analysis were performed to obtain comprehensive transcriptomic data. A total of 17793 genes were detected and GO enrichment analysis was performed on the differentially expressed genes to identify significantly up- and downregulated Biological Process (BP) GO terms across each comparison.

**Results:**

The upregulation of apoptosis-regulating genes and genes related to circulatory system development suggest an induction of these processes, while the downregulation of neurogenesis and gliogenesis genes points to reciprocal regulation during osteogenic differentiation of canine ADSCs.

**Discussion:**

These findings underscore the potential of ADSCs in bone regeneration and offer valuable insights for advancing tissue engineering, however further studies, including proteomic analyses, are needed to confirm these patterns and their biological significance.

## Introduction

Adipose-derived stem cells (ADSCs) are becoming increasingly prominent in regenerative medicine due to their versatility and therapeutic potential (Ong et al., 2021; Feisst et al., 2015). ADSCs can differentiate into various cell types upon *in vitro* induction, including adipocytes, chondrocytes, and osteoblasts, and function as immunomodulators, aiding in migrating immune cells to damaged tissues (Andrzejewska et al., 2019; Ceccarelli et al., 2020). Their high proliferation rate, excellent differentiation capabilities, and ease of extraction with low donor site morbidity make ADSCs attractive for numerous treatments, especially for muscle and bone disorders.

ADSCs can be obtained from both white and brown adipose tissues, with subcutaneous fat being a particularly rich source accessible through minimally invasive procedures like power-assisted liposuction (Palumbo et al., 2018; Bajek et al., 2017). Methods to harvest and culture ADSCs typically involve enzymatic digestion and specific culture conditions to maximize yield. Lipoaspirate obtained via liposuction is a preferred method due to the high number of viable ADSCs it provides. Despite the lack of a standardized approach, these techniques ensure a robust supply of ADSCs for therapeutic applications. Compared to bone marrow-derived mesenchymal stem cells (BM-MSCs), which are limited in number and harder to retrieve, ADSCs offer a more abundant and easily accessible source with lower harvesting risks, positioning them as a viable alternative for mesenchymal stem cell (MSC) therapies (Romero et al., 2017; Francis et al., 2018). The differentiation potential of ADSCs into tissues is well-documented, but the molecular mechanisms underlying their osteogenesis are not fully understood (Jankowski et al., 2020).

Understanding the pathways and mechanisms involved in ADSC differentiation is crucial for optimizing their therapeutic applications. Investigating gene expression and related molecular processes offers deeper insights into how ADSCs differentiate and how these processes can be fine-tuned for regenerative medicine. To fully exploit ADSCs in clinical applications, it is imperative to understand the influence of *ex vivo* conditions and induced differentiations. *Ex vivo* manipulation of ADSCs, involving specific environmental cues and growth factors, plays a pivotal role in directing their differentiation pathways (Durandt et al., 2019; Mende et al., 2021). However, the complexity of these processes often introduces variability in the differentiation outcomes (Bunnell, 2021). Maximizing the therapeutic potential of ADSCs requires meticulous control and standardization of these *ex vivo* conditions, such as culture media composition, oxygen levels, mechanical forces, and the timing of gene expression changes during differentiation (Neri et al., 2013).

Canine ADSCs represent a promising model in stem cell research due to their biological similarities to human ADSCs, including comparable cellular characteristics (e.g., the expression of surface markers such as CD90, CD105 and CD73) and ability to differentiate towards osteogenic, chondrogenic and adipogenic lineages (de Bakker et al., 2013) In addition, these cells are widely accessible and canine models are increasingly recognized in translational research as they bridge the gap between rodent studies and human applications (Merlo and Iacono, 2023). Since dogs are companion animals, sharing the environment and living conditions with their owners, they tend to develop similar diseases to those occurring in humans, such as osteoarthritis or bone fractures (Hoffman and Dow, 2016). Therefore, canine models seem to be relevant in terms of possible therapeutic approaches not only in veterinary medicine, but also in humans.

A comprehensive understanding of the molecular processes governing ADSC osteogenesis, including gene expression and regulatory pathways, and their responses to varying *ex vivo* conditions and differentiation procedures, is critical for translating ADSC-based therapies from the laboratory to clinical practice. Ensuring the reproducibility and predictability of these conditions is vital to unlocking the full therapeutic potential of ADSCs in treating bone diseases and injuries. Thus, this study focused on functional gene expression analysis in canine ADSCs subjected to long-term culture and osteogenic differentiation. Bioinformatic analysis provided a broad view of the impact of *ex vivo* conditions and induced differentiation on ADSCs, offering valuable molecular insights. The knowledge gained serves as a reference for understanding ADSC-related processes and their application in bone regeneration therapies.

## Materials and methods

### Material source and cell cultures

ADSCs were isolated from canine omental adipose tissue samples obtained from waste material discarded from routine surgeries. Due to the sourcing of research material from surgical waste, without subjecting the animals to any additional procedures, the study was exempt from obtaining Bioethical Committee Approval. The waste material was derived from healthy, young (<1-year-old) female dogs of varying breeds, subjected to ovariohysterectomy sterilization surgery soon after the end of the first estrous cycle. Specimens of the same sex, similar age, and similar estrous status were chosen to minimize the effect of hormonal differences on the obtained stem cells' biology and differentiation (Gavin and Bessesen, 2020; Steiner and Berry, 2022). The material was stored in sterile PBS at 4°C immediately after collection, and transported into the cell culture laboratory after no longer than 24 h. It was then homogenized using a sterile surgical blade, and subjected to enzymatic digestion using a 1 mg/mL collagenase II solution. The resulting cells were collected in 50 mL falcon tubes using centrifugation, suspended in DMEM supplemented with 10% FBS, 4 mM of L-glutamine, and 1x antibiotic-antimycotic solution. Samples from the resulting cell suspension were subjected to morphological and cytometric evaluation to confirm their ADSC identity, as described in detail in our previous work (Jankowski et al., 2021; Jankowski et al., 2022). The initial ADSC identity was assessed based on their mesenchymal stem cell morphology, as well as the expression of CD44 and CD90, and the lack of CD45 and CD34 expression. Each biological sample was simultaneously seeded onto three wells of a six-well culture plate, with one serving as a source of cells for further RNA isolation, the other subjected to staining to determine the success of the differentiation or, in the case of late control samples, serve as staining control, and the third one collected after 1 day of culture as an early control. ADSCs were differentiated into osteoblasts over 14 days (OT) and compared to controls: ADSCs cultured for 14 days without differentiation medium (OC) and ADSCs cultured for 1 day (EC). The cultures were monitored every day, with signs of abnormal morphology, lack of signs of proliferation and/or differentiation, and any signs of microbial infection disqualifying the culture from further studies. The success of differentiation was determined based on successful Alizarin Red (A5533, Sigma-Aldrich, Saint Louis, MO, United States) staining. The cultures for further RNA isolation were selected based on their final morphology, the results of staining, and the visual quality of the culture medium, with only the cultures that exhibited satisfactory characteristics in all of these aspects used for further steps of the study. At all the analyzed time points, cell culture samples were collected and stored in 1 mL of TRIzol (Thermo-Fischer Scientific, Waltham, MA, United States) at −80°C. RNA was isolated from the samples using a commercially sourced kit (TRIzol Plus Purification KIT, 12183555, Thermo-Fischer Scientific, Waltham, MA, United States). The parameters of the isolated RNA were then analyzed using the NanoDrop spectrophotometer (Thermo-Fischer Scientific, Waltham, MA, United States), with only the samples with sufficient RNA quantity (>50 ng/μL) and quality (260/280 Ratio >1.9) selected for further analysis. Furthermore, upon arrival to the sequencing facility, the samples were once again evaluated to ensure appropriate yields and quality. The results showed an average RNA concentration of 107,38 ± 29,38 ng/μL and RIN of 9,64 ± 0,28, with none of the samples exhibiting RIN < 9.3. Each time point was analyzed in triplicate (3 biological samples from different canine specimens to minimize the effect of inter-specimen differences in expression). In total, 9 biological samples (3 early control, 3 late control, and 3 differentiated osteoblasts) were subjected to RNA sequencing (RNA-seq), performed by an experienced contractor (CeGaT GmbH, Tübingen, Germany) using the Illumina platform (Illumina, San Diego, CA, United States), to obtain comprehensive transcriptomic data. All the data obtained from the RNAseq analysis was deposited to the publicly available Gene Expression Omnibus (GEO) repository (accession nr: GSE280031). The detailed cell culture, identity assessment (confirming the success of osteogenic differentiation), RNA isolation, and RNA-seq methodology were described in previous publications of our team (Jankowski et al., 2021; Jankowski et al., 2022).

### Bioinformatical analysis

All analyses were performed in R (version 4.3.2). Raw count data and normalized count data were imported into R for analysis using the readr pachage. Raw count data from two TSV files were merged based on gene identifiers annotated using org.Cf.e.g.,.db (part of Bioconductor project accessed via BiocManager) (Gentleman et al., 2004), using dplyr and tidyr for data manipulation. 

Differential gene expression analysis was performed using the DESeq2 package (Love et al., 2014). In particular, the data processing and filtering pipeline consisted of multiple steps to ensure robust differential expression analysis and meaningful enrichment results. Initially, raw counts were filtered to retain genes with counts greater than or equal to 10 in at least three samples, removing low-expressed genes unlikely to be biologically relevant. DESeq2 preprocessing involved normalization to correct for library size differences and dispersion estimation to model variance accurately. Differential expression was assessed using contrasts between experimental conditions, with significance defined by an adjusted *p*-value <0.05 to control the false discovery rate. Genes with log2 fold change ≥1 or ≤ -1 were further selected to focus on biologically significant changes. As a part of DESeq2 analysis, artifacts were mitigated through independent filtering to exclude low-power genes and empirical Bayes shrinkage to handle outliers.

Variance stabilizing transformation (VST) was applied to the count data, followed by principal component analysis (PCA) to explore sample groupings based on experimental conditions. PCA results were visualized using ggplot2. Volcano plots highlighting overall differential expression patterns were generated with EnhancedVolcano, while tree plots illustrating the clustering of GO terms were created using Enrichplot.

GO enrichment analysis for Biological Processes (BP), Cellular Component (CC) and Molecular Function (MF) was conducted separately for upregulated and downregulated genes using clusterProfiler (Wu et al., 2021), and results were visualized with bar plots showing average log2 fold change by GO term. Furthermore, KEGG pathway enrichment analysis was performed using the clusterProfiler package to identify significantly enriched pathways associated with differentially expressed genes. The analysis was conducted for each comparison of interest, with adjusted *p*-values below 0.05 considered significant. Enriched pathways were visualized using map with vertices denoting the pathways and edges between them. In this plot pathway relationships (edges) were quantified using pairwise similarity scores based on gene overlap. This approach enabled the identification of clusters of functionally related pathways, highlighting key biological processes and molecular interactions.

## Results

**FIGURE 1:**
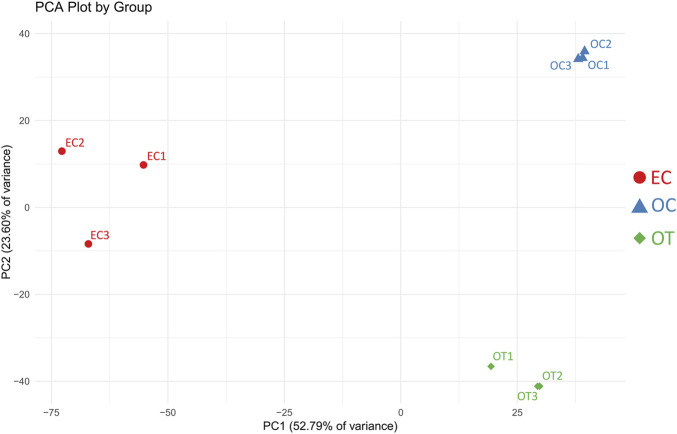
PCA results of the analyzed samples. EC- early control (24 h), OC-late control (14 days), OT-differentiated osteoblasts. The *x*-axis represents the first principal component (PC1), explaining 52.79% of the variance, while the *y*-axis represents the second principal component (PC2), explaining 23.60% of the variance. Samples within the same group cluster together, indicating similar expression patterns, while distinct separation between groups reflects differences in gene expression profiles.

The PCA of the samples analyzed in the study demonstrated that the biological repetitions of the three studied time points form distinct clusters. This initially confirms the relatively similar gene expression among the studied sample groups (EC-early control, 1 day of culture; OC-osteoblast control, 14 days of culture without differentiating medium; OT-osteoblast test, 14 days of culture with differentiating medium), and major differences in gene expression between the groups. The results of the PCA are presented in Figure 1.

**FIGURE 2:**
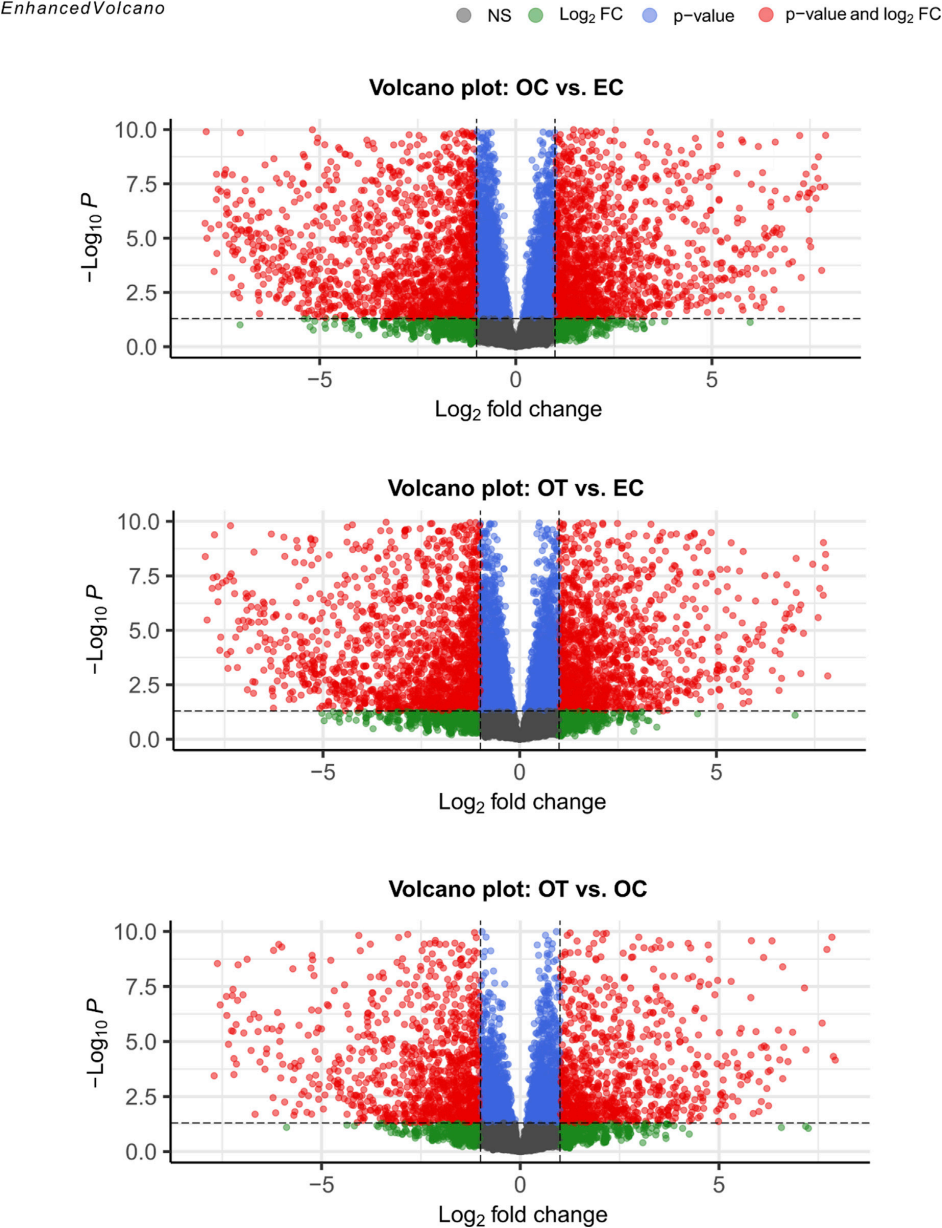
Volcano plots visualization of differential gene expression analysis. The red points denote genes that met the conditions for differential gene expression (Log_2_FC ≥ 1 and FDR < 0.05). Green points indicate genes that met the fold change criterion but did not meet significance conditions, while blue points mark genes that only met the latter. Finally, grey points indicated genes that did not meet any of the DEG conditions.

A total of 17793 genes were detected in the RNAseq analysis. Differential expression analysis, using a threshold of Log_2_FC ≥ 1 and FDR<0.05, yielded 1736 DEGs between OC and EC, 1471 DEGs between OT and EC, and 722 genes between OT and OC. The results of the differential expression analysis were presented as volcano plots, compiled in Figure 2. Furthermore, the complete list of DEGs, including their LogFCs and *p*-values, were included in the manuscript as Supplementary Table 1.

**FIGURE 3:**
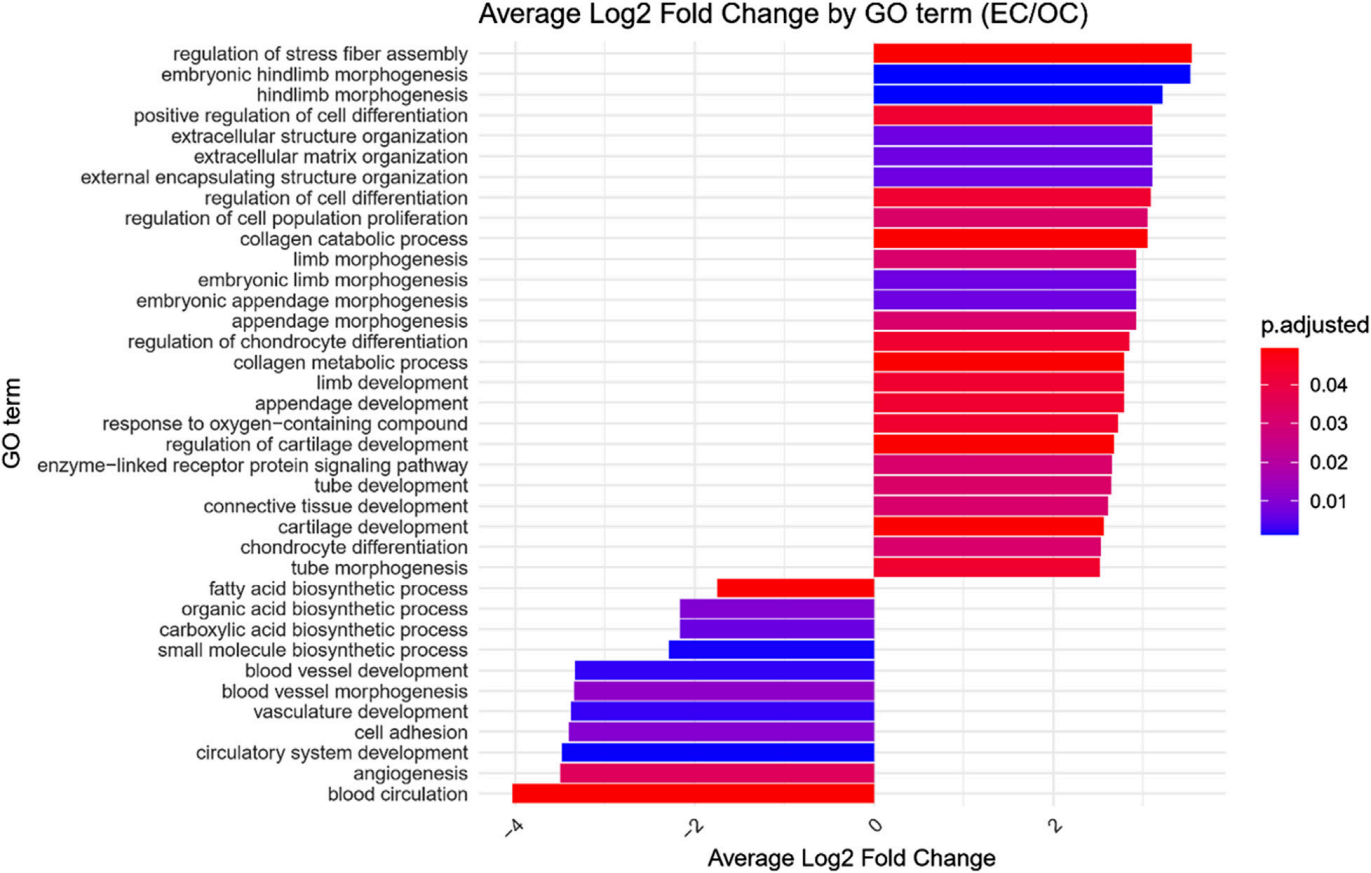
Bar graph presenting GO terms differentially enriched between the early control (24 h) and osteoblast control (14 days) cultures.The size of the bars represent the average fold-change of differentially expresed genes in the enriched groups, while the color of the bar represents the adjusted *p*-value of the GO enrichment (red-higher, blue-lower).

**FIGURE 4:**
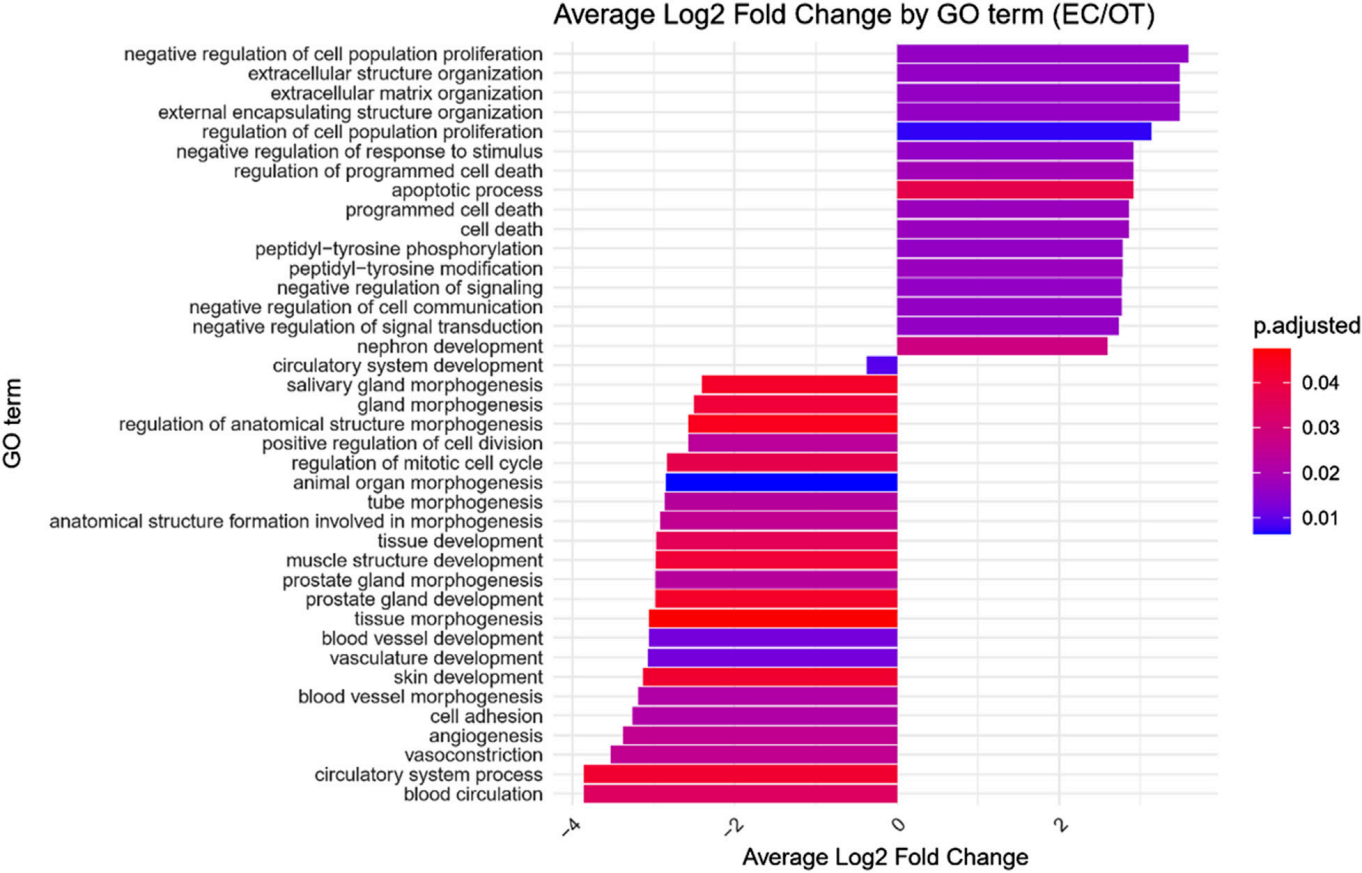
Bar graph presenting GO terms differentially enriched between the early control (24 h) and differentiated osteoblast cultures. The size of the bars represent the average fold-change of differentially expresed genes in the enriched groups, while the color of the bar represents the adjusted *p*-value of the GO enrichment (red-higher, blue-lower).

**FIGURE 5:**
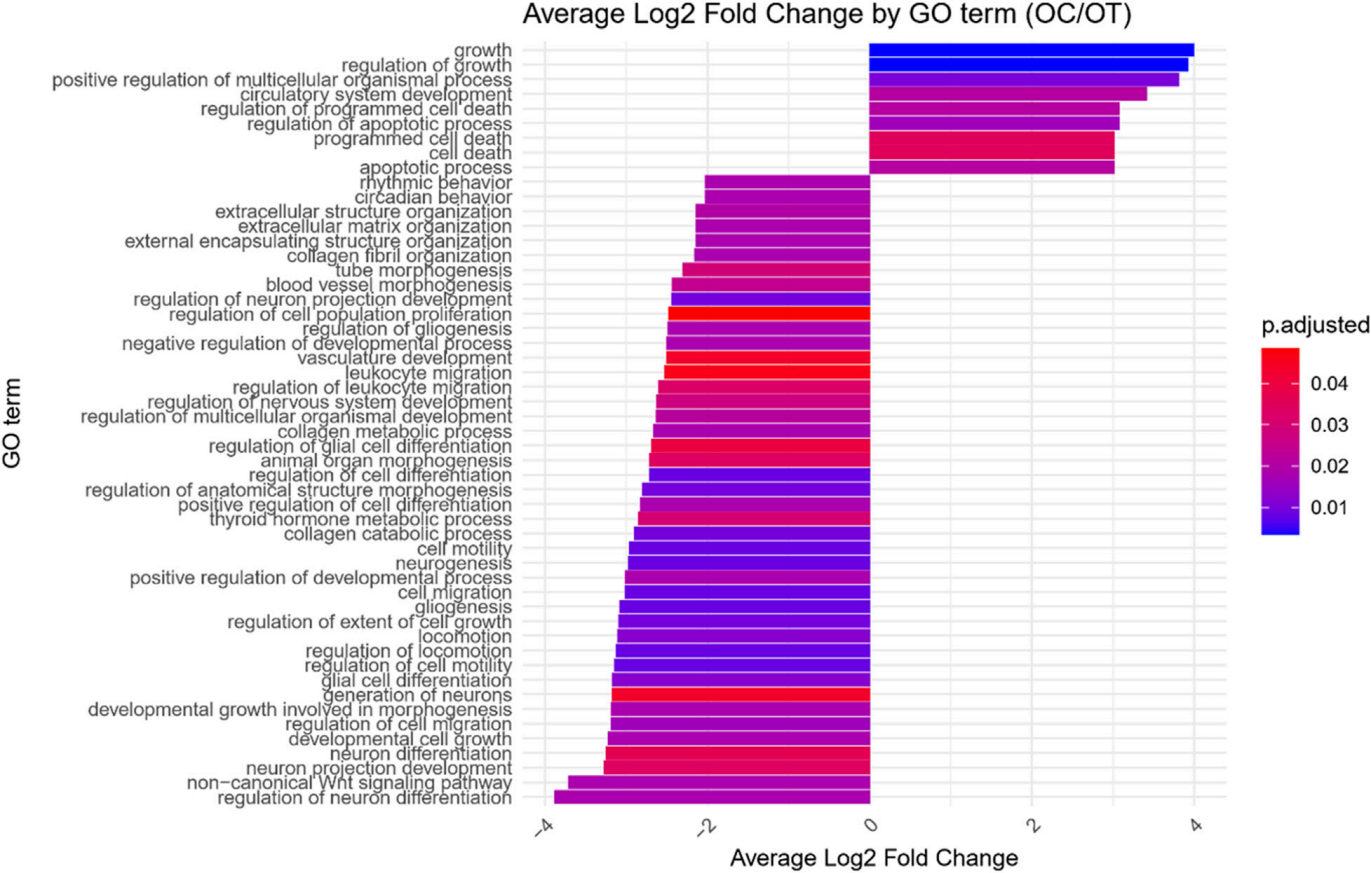
Bar graph presenting GO terms differentially enriched between the osteoblast control (14 days) and differentiated osteoblast cultures. The size of the bars represent the average fold-change of differentially expresed genes in the enriched groups, while the color of the bar represents the adjusted *p*-value of the GO enrichment (red-higher, blue-lower).

GO enrichment analysis was performed on the differentially expressed genes to identify significantly over- and under-represented Biological Process (BP), Cellular Component (CC) and Molecular Function (MF) GO terms across each comparison. The BP category was chosen as the focus of this study, as it provides a comprehensive view of the functional dynamics underlying osteogenic differentiation. Nonetheless, the complete results of GO enrichment analysis were included in the manuscript as Supplementary Table 2. The analysis revealed 26 significantly over-represented and 11 under-represented GO BP terms in the early control vs osteoblast control comparison, 16 over- and 23 under-represented terms in the early control vs differentiated osteoblast comparison, and 9 over- and 43 under-represented terms in the osteoblast control vs differentiated osteoblast comparison. The results of the GO BP enrichment analysis are visualized as bar graphs in Figures 3–5.

**FIGURE 6:**
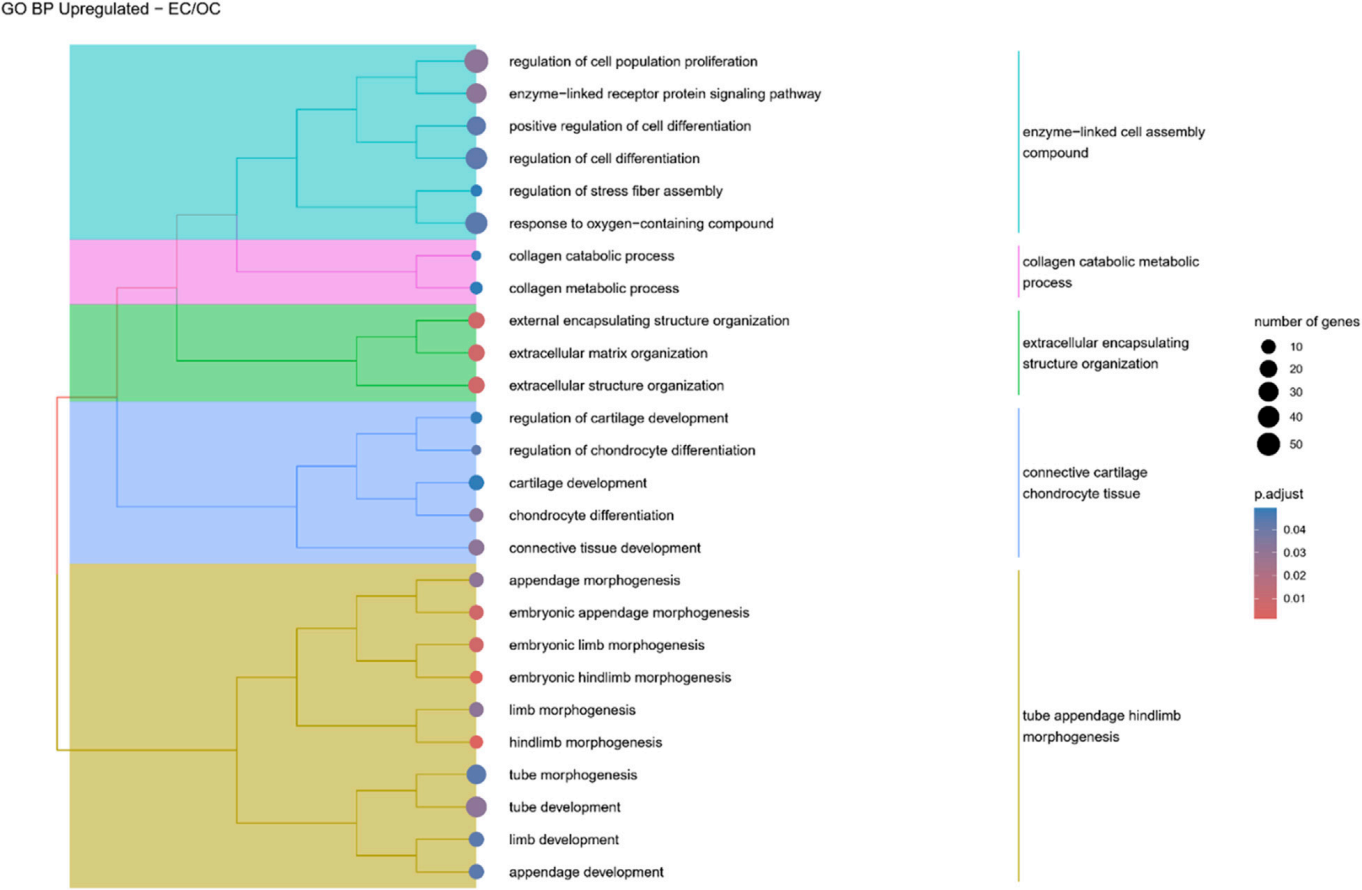
A tree plot presenting the results of hierarchical clustering of the GO terms positively enriched between Early control (24 h) and osteoblast control (14 days) culture. The GO terms were clustered based on the data contained in the GO database, with larger clusters marked with different colors. The size of the circle next to the GO name represents the number of differentially expressed genes present in this GO, while the color marks the *p*-value of the GO enrichment (blue-higher, red-lower). The clusters are annotated with keywords that most often repeat in the GO terms contained within).

**FIGURE 7:**
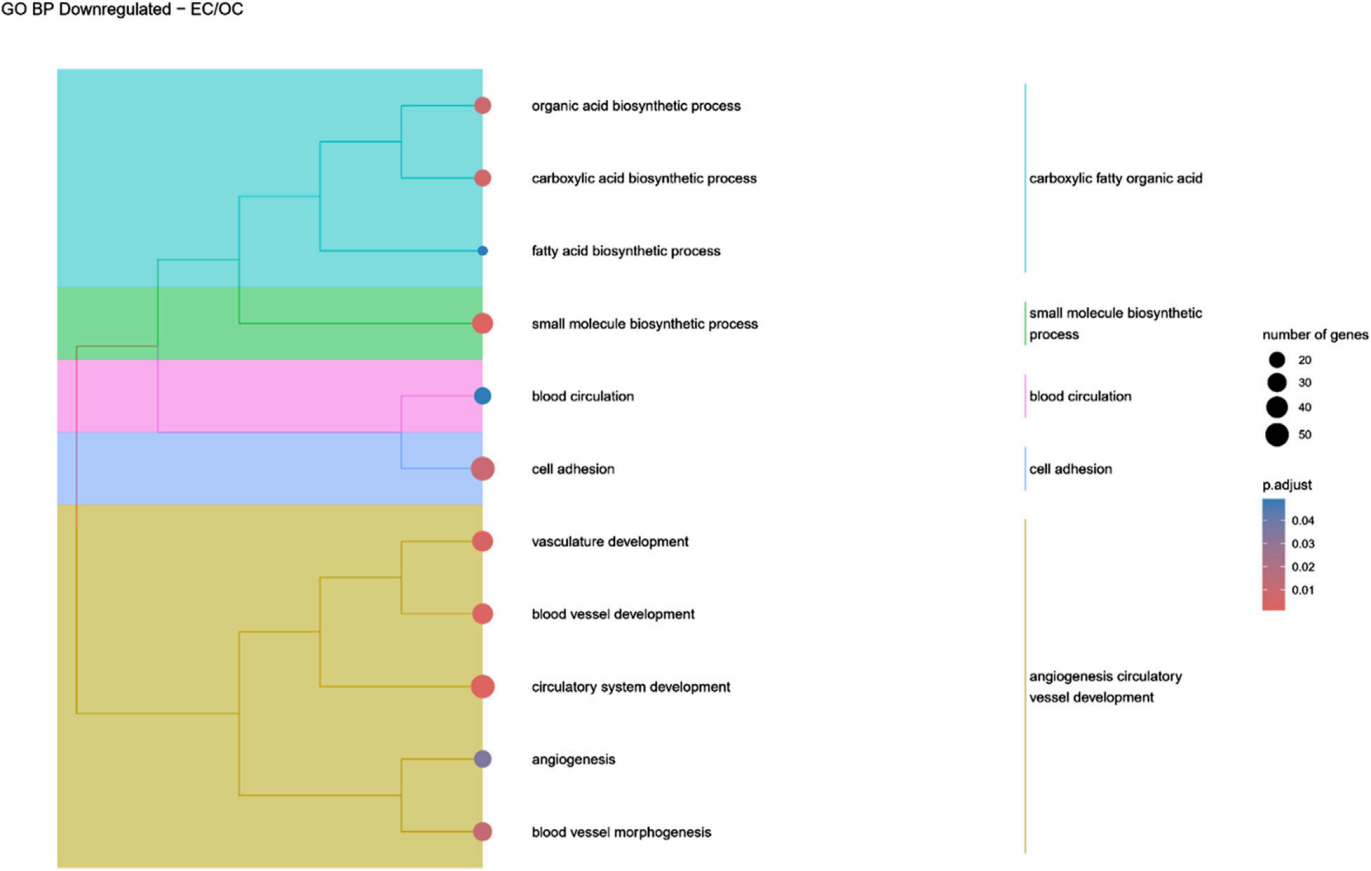
A tree plot presenting the results of hierarchical clustering of the GO terms negatively enriched between Early control (24 h) and osteoblast control (14 days) culture. The GO terms were clustered based on the data contained in the GO database, with larger clusters marked with different colors. The size of the circle next to the GO name represents the number of differentially expressed genes present in this GO, while the color marks the *p*-value of the GO enrichment (blue-higher, red-lower). The clusters are annotated with keywords that most often repeat in the GO terms contained within).

**FIGURE 8:**
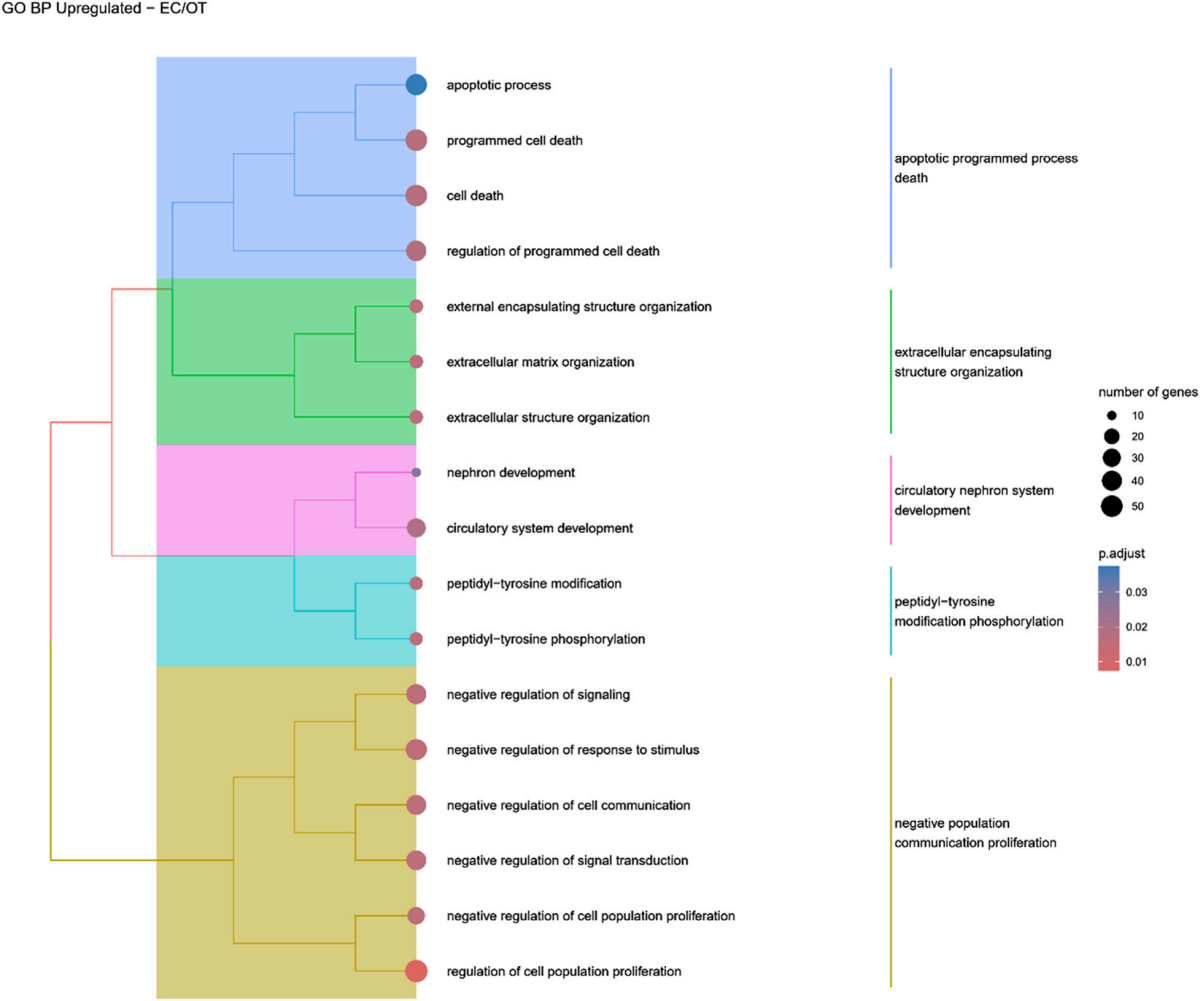
A tree plot presenting the results of hierarchical clustering of the GO terms positively enriched between early control (24 h) and differentiated osteoblast (14 days) culture. The GO terms were clustered based on the data contained in the GO database, with larger clusters marked with different colors. The size of the circle next to the GO name represents the number of differentially expressed genes present in this GO, while the color marks the *p*-value of the GO enrichment (blue-higher, red-lower). The clusters are annotated with keywords that most often repeat in the GO terms contained within).

**FIGURE 9:**
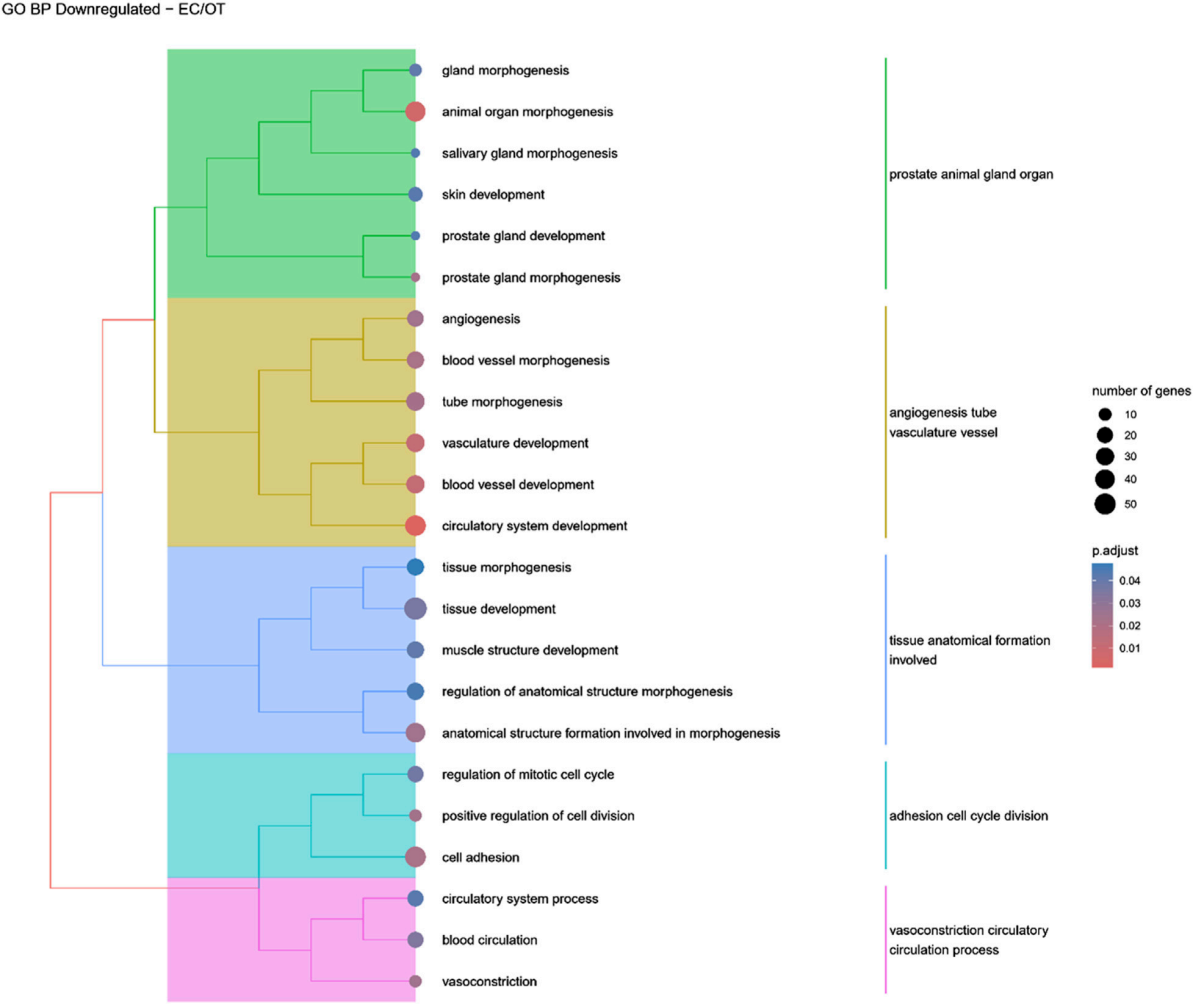
A tree plot presenting the results of hierarchical clustering of the GO terms negatively enriched between early control (24 h) and differentiated osteoblast (14 days) culture. The GO terms were clustered based on the data contained in the GO database, with larger clusters marked with different colors. The size of the circle next to the GO name represents the number of differentially expressed genes present in this GO, while the color marks the *p*-value of the GO enrichment (blue-higher, red-lower). The clusters are annotated with keywords that most often repeat in the GO terms contained within).

**FIGURE 10:**
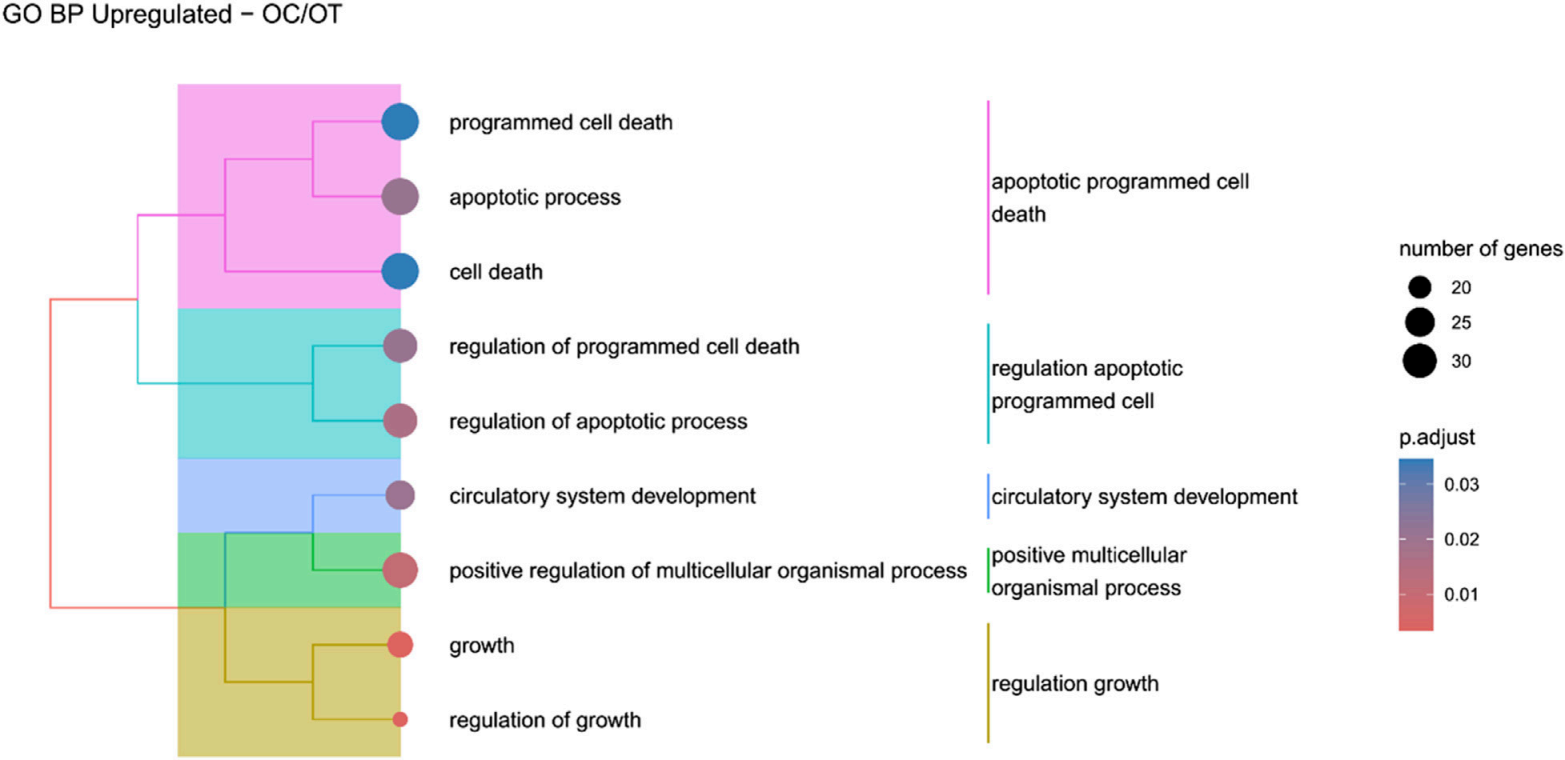
A tree plot presenting the results of hierarchical clustering of the GO terms positively enriched between osteoblast control (14 days) and differentiated osteoblast (14 days) culture. The GO terms were clustered based on the data contained in the GO database, with larger clusters marked with different colors. The size of the circle next to the GO name represents the number of differentially expressed genes present in this GO, while the color marks the *p*-value of the GO enrichment (blue-higher, red-lower). The clusters are annotated with keywords that most often repeat in the GO terms contained within).

**FIGURE 11:**
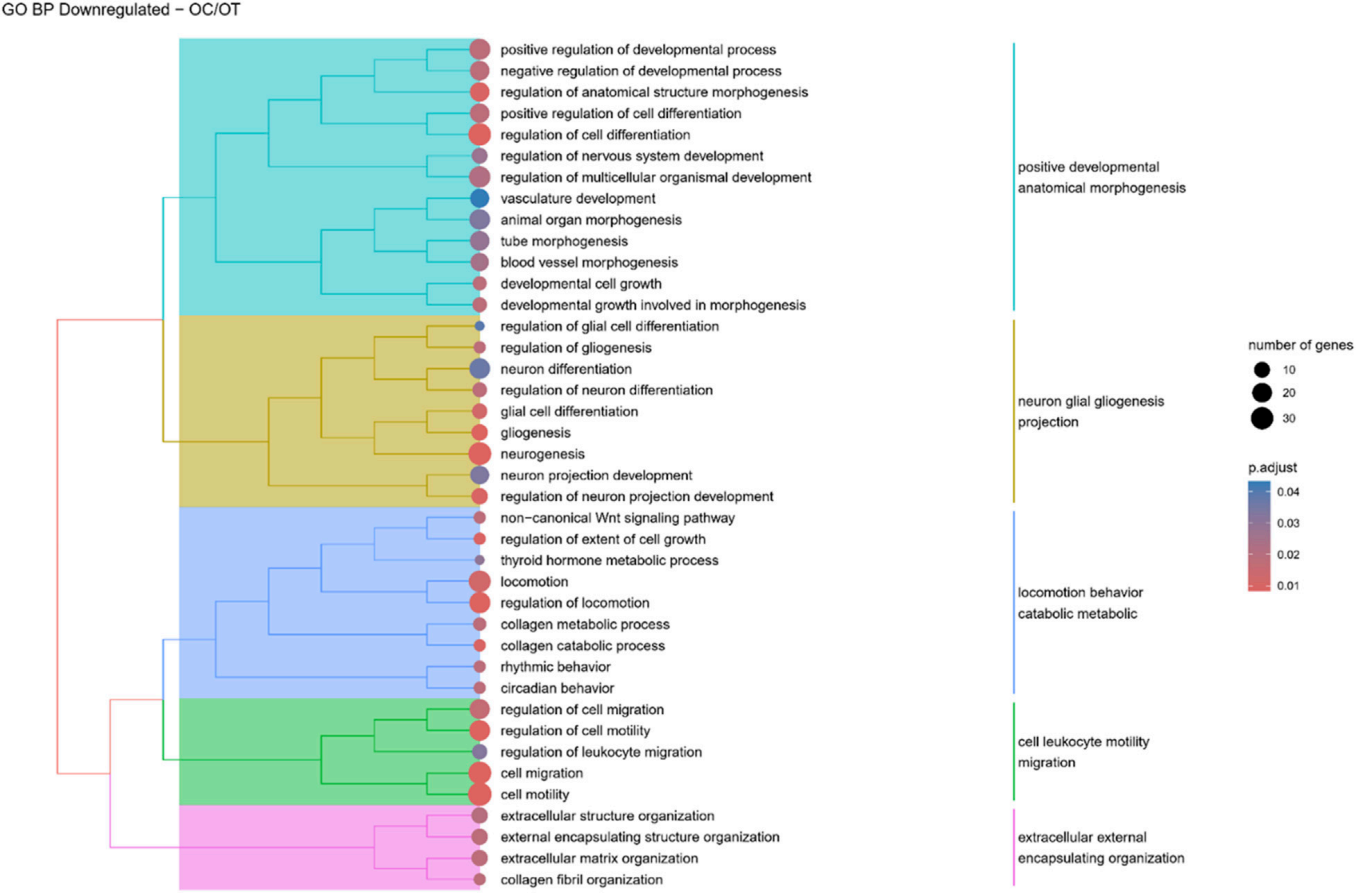
A tree plot presenting the results of hierarchical clustering of the GO terms negatively enriched between osteoblast control (14 days) and differentiated osteoblast (14 days) culture. The GO terms were clustered based on the data contained in the GO database, with larger clusters marked with different colors. The size of the circle next to the GO name represents the number of differentially expressed genes present in this GO, while the color marks the *p*-value of the GO enrichment (blue-higher, red-lower). The clusters are annotated with keywords that most often repeat in the GO terms contained within).

Hierarchical clustering of GO terms revealed distinct groups of significantly over- and under-represented biological processes in each comparison. In the early control vs osteoblast control comparison, processes such as enzyme-linked cell assembly, collagen catabolic process, extracellular matrix organization, connective cartilage development, and hindlimb morphogenesis were notably positively enriched (Figure 6). Conversely, processes related to carboxylic acid and fatty acid metabolism, small molecule biosynthesis, blood circulation, cell adhesion, and angiogenesis were significantly negatively enriched (Figure 7). In early control vs differentiated osteoblasts comparison, positively enriched groups included apoptotic programmed process death, extracellular encapsulating structure organization, circulatory nephron system development, peptidyl-tyrosine modification phosphorylation, and negative population communication proliferation (Figure 8). In the same comparison, negatively enriched groups comprised prostate animal gland organ, angiogenesis tube vasculature vessel, tissue anatomical formation involved, adhesion cell cycle division, and vasoconstriction circulatory circulation process (Figure 9). In the osteoblast control vs differentiated osteoblast comparison, over-represented processes involved apoptotic programmed cell death, regulation of apoptotic programmed cell death, circulatory system, positive multicellular organismal process, regulation growth (Figure 10). Meanwhile, under-represented processes included positive developmental anatomical morphogenesis, neuron glial gliogenesis projection, locomotion behavior catabolic metabolic, cell leukocyte motility migration, and extracellular external encapsulation organization (Figure 11).

**FIGURE 12:**
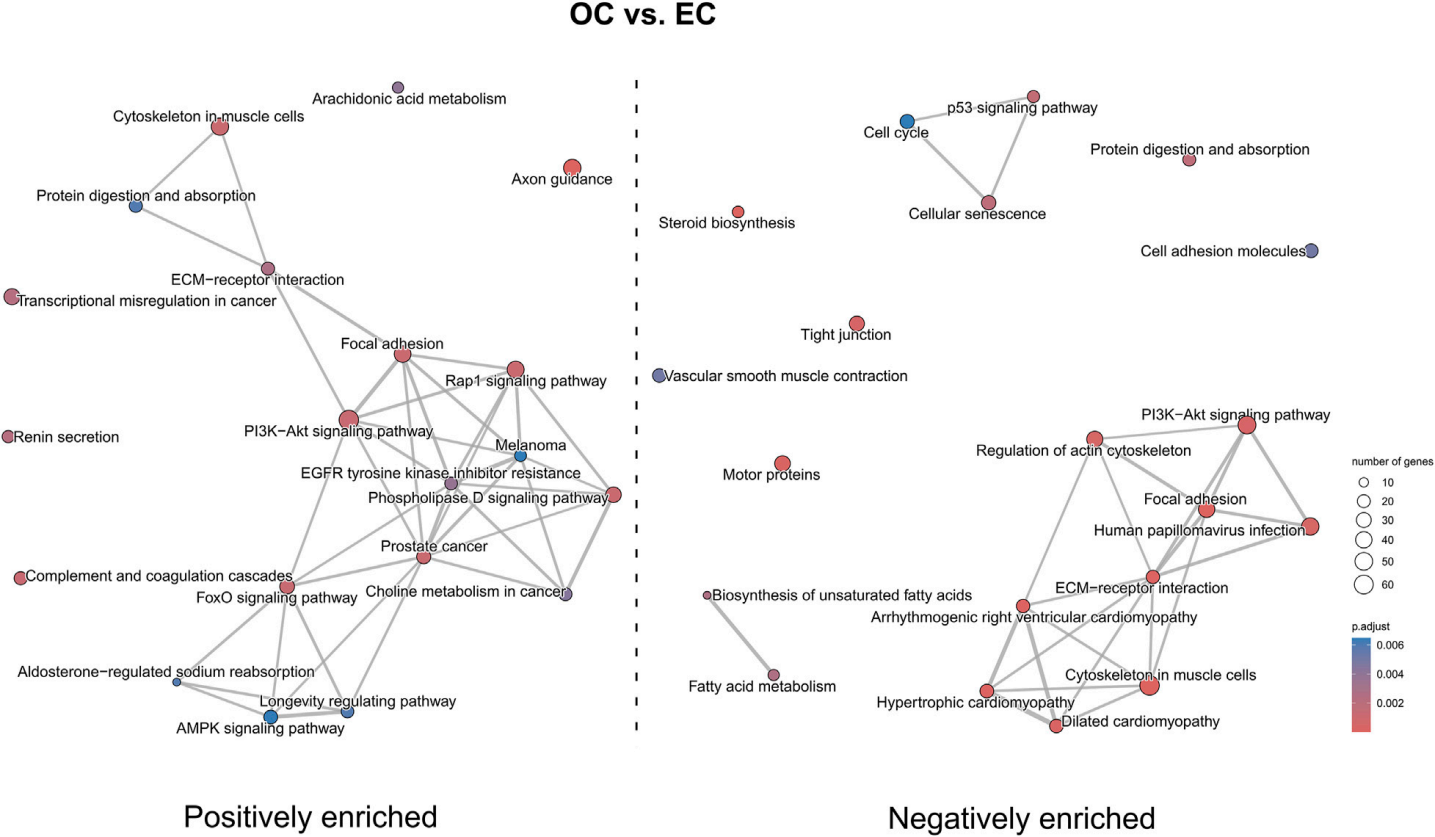
The map of KEGG pathways differentially enriched between the early control and late control cultures. The circles on the map represent enriched pathways, with their size indicating the number of differentially expressed genes contained in the pathway, and the color denoting the adjusted *p*-value of the enrichment (blue-higher, red-lower). The lines connecting the pathway indicate the presence of common genes between the connected pathways, with a thicker line representing a bigger number of genes in common.

Finally, the KEGG pathway enrichment analysis revealed significantly enriched pathways associated with differentially expressed genes. In OC *versus* EC (Figure 12) comparison pathways such as “axon guidance” and “AMPK signaling pathway” were positively enriched, while “p53 signaling pathway”, “hypertrophic cardiomyopathy” and “regulation of actin cytoskeleton” were negatively enriched. Pathways such as “PI3K-Akt signaling pathway”, “focal adhesion” and ‘ECM-receptor interaction’ were both positively and negatively enriched.

**FIGURE 13:**
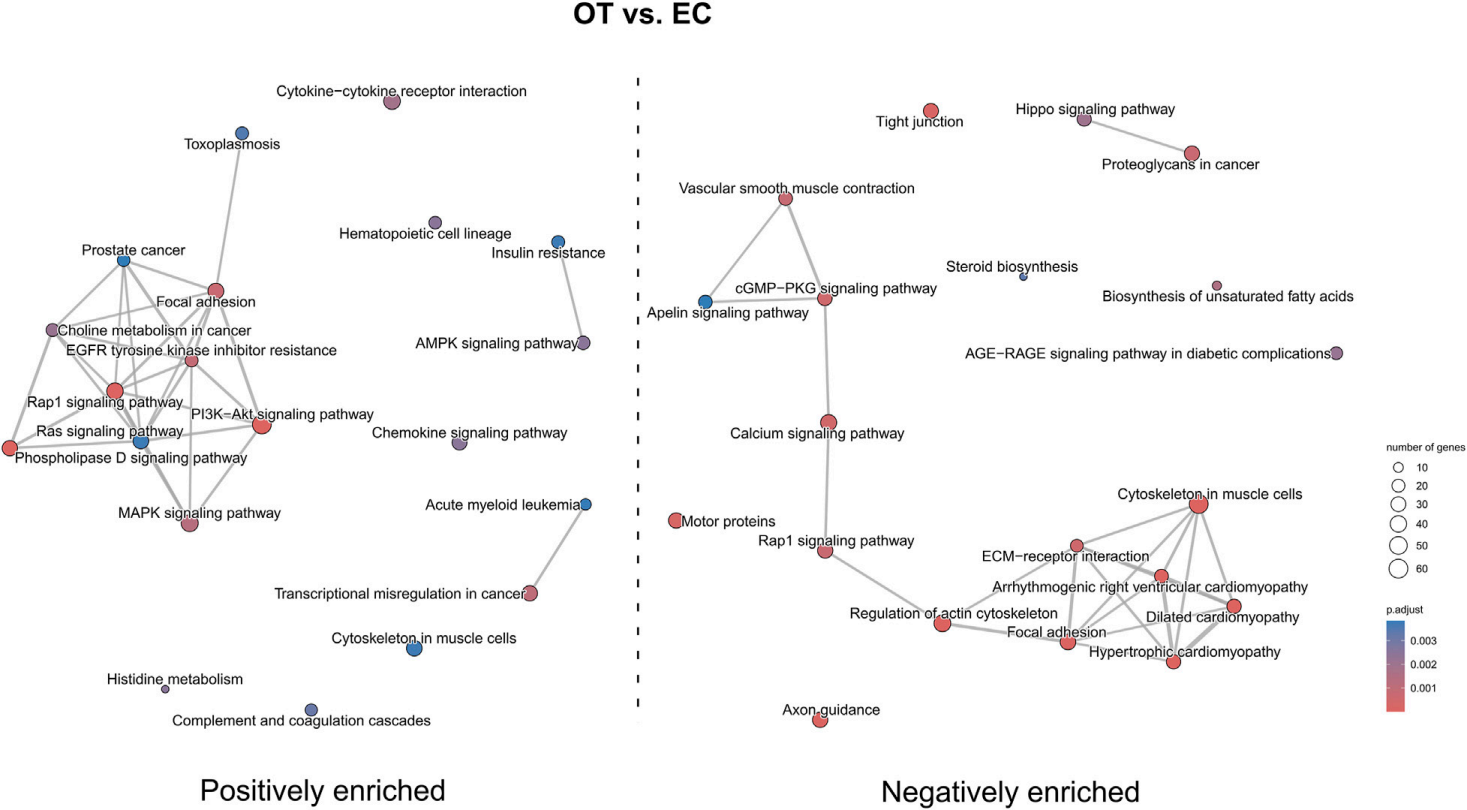
The map of KEGG pathways differentially enriched between the early control and differentiated osteoblast cultures. The circles on the map represent enriched pathways, with their size indicating the number of differentially expressed genes contained in the pathway, and the color denoting the adjusted *p*-value of the enrichment (blue-higher, red-lower). The lines connecting the pathway indicate the presence of common genes between the connected pathways, with a thicker line representing a bigger number of genes in common.

In the OT *versus* EC comparison (Figure 13), a positive enrichment of pathways such as “Ras signaling pathway”, “PI3K-Akt signaling pathway”, “AMPK signaling pathway”, and “focal adhesion” were observed, while pathways like “axon guidance”, “hypertrophic cardiomyopathy”, “ECM-receptor interaction” and “tight junction” were negatively enriched. Notably, “Rap1 signaling pathway” was both positively and negatively enriched.

**FIGURE 14:**
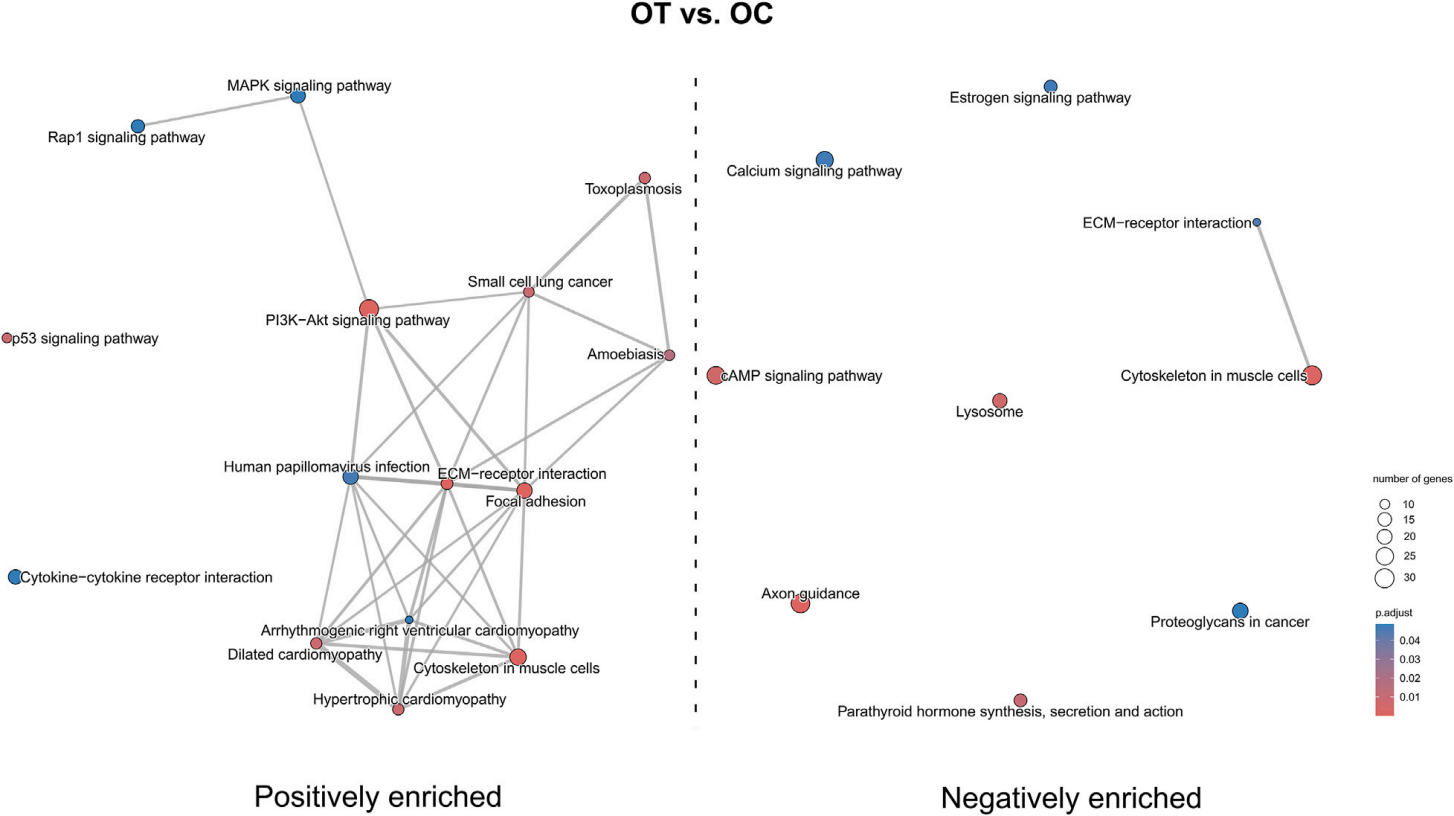
The map of KEGG pathways differentially enriched between the late control and differentiated osteoblast cultures. The circles on the map represent enriched pathways, with their size indicating the number of differentially expressed genes contained in the pathway, and the color denoting the adjusted *p*-value of the enrichment (blue-higher, red-lower). The lines connecting the pathway indicate the presence of common genes between the connected pathways, with a thicker line representing a bigger number of genes in common.

In the OT *versus* OC comparison (Figure 14) pathways such as “Rap1 signaling pathway”, “MAPK signaling pathway”, “PI3K-Akt signaling pathway”, “p53 signaling pathway”, “hypertrophic cardiomyopathy” and “focal adhesion” were positively enriched, while “calcium signaling pathway”, “axon guidance”, “cAMP signaling pathway” and “lysosome” were negatively enriched. “ECM-receptor interaction” was both positively and negatively enriched.

## Discussion

In the present study, the RNA-seq analysis was performed to shed light on biological processes involved in osteogenic differentiation of canine ADSCs. It is important to note that *in vitro* osteogenic differentiation typically requires at least 14 days, meaning the differentiated cells are exposed to prolonged culture conditions, which may impact their metabolism. To determine transcriptomic changes in differentiated ADSCs occurring only due to the differentiation process, a comparative was performed between ADSCs cultured for 14 days without differentiation medium (osteoblast control, OC) and ADSCs cultured for only 1 day (early control, EC). RNA-seq, a high-throughput technique that enables comprehensive transcriptome analysis, was used due to its reproducibility and effectiveness in identifying transcriptomic markers of osteogenic differentiation (Wang et al., 2009). Gaining further insight into the molecular changes in cultured and differentiated ADSCs is essential before considering their clinical applications. To provide a deeper understanding of the functional changes during osteogenic differentiation, we performed a pathway enrichment analysis, which allowed us to identify key molecular pathways driving the observed biological processes.

‘PI3K-Akt signaling pathway’ was both positively and negatively enriched in OC *versus* EC comparison, while its over-representation was observed in OT *versus* EC and OT *versus* OC comparisons. This pathway was already demonstrated to play crucial role in osteogenic differentiation of MSCs. In case of umbilical cord-derived MSCs (UC-MSCs) this pathway was positively enriched after the 3- and 5-day osteogenic differentiation period, while its inhibition led to the blockage of the differentiation (Gao et al., 2023). Additionally, Runx2, a key transcription factor for osteogenic differentiation was shown to work in conjunction with this pathway, promoting both osteoblast and chondrocyte differentiation and migration (Fujita et al., 2004). The current results are consistent with previous studies and suggest that ‘PI3K-Akt signaling pathway’ promotes osteoblast lineage commitment and maturation in the presence of osteogenic supplements after 14-day differentiation period in ADSCs.

The comparison of osteogenically differentiated ADSCs and both EC and OC revealed the upregulation of genes associated with apoptosis and its regulation. Notably, in the OC/OT comparison, five out of nine over-represented GO groups were related to cell death. It can be hypothesized that this was not due to the prolonged culture, as the osteoblast control, which was cultured for the same duration, did not show an increase in apoptosis-related gene expression compared to EC. Apoptosis plays a crucial role in maintaining bone homeostasis by balancing tissue renewal and degradation, which is essential for continuous bone remodelling. In addition, stem cells rely on robust quality control mechanisms to preserve their molecular integrity over extended durations. One critical aspect of this quality control involves initiating cell death damaged cells (Reya et al., 2001). Key regulators of apoptosis include members of the BCL2 protein family, which modulate cell survival depending on the BCL2/BAX ratio (Youle and Strasser, 2008). The roles of BCL2 and BCL2L1 (also known as BCL-XL) have been previously documented in the osteogenic, adipogenic, and neurogenic differentiation of human BM-MSCs. BCL2L1 was found to be expressed at consistent levels in both undifferentiated and differentiated BM-MSCs, where it exhibited anti-apoptotic activity. In contrast, BCL2 expression was dependent on the stage of differentiation and increased only in differentiated cells (Oliver et al., 2011). Similarly, in adipo- and osteo-induced dental pulp MSCs, BCL2 was also overexpressed (Lee et al., 2019). In this study, BCL2L1 was upregulated in osteo-induced canine ADSCs, suggesting the inhibition of apoptosis in these cells during osteogenic differentiation.

There are conflicting results regarding the expression of apoptosis-related genes in MSCs after differentiation. Similarly to our findings, Robert et al. (2018) observed an over-representation of apoptosis-regulation GO groups during the early stages of osteogenic differentiation in human ADSCs. Likewise, increases in apoptosis-related gene expression have been reported following osteogenic, chondrogenic, adipogenic, and neurogenic differentiation of human Wharton’s jelly-derived MSCs (WJ-MSCs) (Stefańska et al., 2023). Similarly, Oliver et al. (2013) reported an increased sensitivity towards apoptosis after adipogenic and osteogenic differentiation of human BM-MSCs, which they attributed to decreased DNA damage repair. On the contrary, other studies, such as by Lo Furno et al. (2013), have reported decreased apoptosis markers after adipogenic differentiation in human ADSCs, indicating that the expression of apoptosis-related genes may vary depending on the MSC source and differentiation stage, which should be carefully considered in future studies.

Additionally, ‘p53 signaling pathway’ was negatively enriched in OC *versus* EC comparison and positively enriched in OT *versus* OC comparison, reflecting its multifaceted role in cellular regulation. p53 encodes a transcription factor involved in major cellular processes, such as cell cycle arrest, cellular senescence and apoptosis (Hernández Borrero and El-Deiry, 2021). Its role in regulation of gene expression associated with mesenchymal differentiation programs, including osteogenic differentiation, has also been demonstrated (Jain and Barton, 2018). The negative enrichment of this pathway in OC *versus* EC suggests reduced stress response and apoptosis during the initial culture phase, while its over-representation in OT *versus* OC indicates the reactivation of this pathway during osteogenic differentiation. Although, studies utilizing murine MSCs revealed that p53 plays an inhibitory role in their osteogenic differentiation (Armesilla-Diaz et al., 2009; Tataria et al., 2006), the current results underscore the need for further investigation into its species-specific and context-depended functions.

Genes involved in “circulatory system development” were also upregulated in osteo-induced ADSCs as compared to EC and OC. However, terms related to “blood vessel morphogenesis” and “vasculature development” were under-represented, while “angiogenesis,” “tube morphogenesis,” “blood vessel development”, and “circulatory system development” were negatively enriched in EC/OT comparison. The connection between osteogenesis and the circulatory system is well-established, as both intramembranous and endochondral bone formation are closely linked to vascular ingrowth. In particular, capillary invasion during intramembranous ossification triggers MSC differentiation into osteoblasts (Kanczler and Oreffo, 2008). Moreover, the development of the circulatory system is crucial for maintaining the homeostasis of bone tissue, as blood vessels supply the nutrients required for osteogenic differentiation (Kanczler and Oreffo, 2008). Previous studies showed that ADSCs differentiate towards endothelial cells and enhance angiogenesis more than BM-MSCs (Cao et al., 2005; Brennan et al., 2017). Shaik et al. (2019) also observed the enrichment of genes associated with ECM organization and angiogenesis along with ERK1/2 and JNK pathways in human ADSCs following a 21-day osteogenic differentiation period, suggesting that angiogenic-related genes may promote vascular development and integration with bone tissue, a trend also observed in canine ADSCs. However, the under-representation of ‘blood vessel morphogenesis’ and ‘vasculature development’ groups might indicate that canine ADSCs during osteogenic differentiation tend to support the existing vessels rather than participate in the formation of new ones.

The pathway enrichment analysis provides a further insight into the dynamic changes of expression of genes associated with circulatory system during osteogenic differentiation. “Hypertrophic cardiomyopathy”, “dilated cardiomyopathy” and “arrhythmogenic right ventricular cardiomyopathy” were positively enriched in OT *versus* OC comparison, while negatively enriched in OT/EC and OC/EC comparisons, suggesting a complex role of these pathways in the progression form multipotent stated to osteogenic differentiation. The activation of cardiomyopathy-associated pathways in OT might indicate their involvement into osteogenic-specific processes, for instance in ECM remodeling or calcium singaling.

In osteoblast controls, genes associated with circulatory system development, as well as with blood vessel development and morphogenesis, angiogenesis, blood circulation, and vasculature development, were downregulated. This likely reflects the limitations of two-dimensional *in vitro* culture, which does not fully replicate the complexity of *in vivo* conditions, reducing the need for advanced developmental processes (Gibler et al., 2021). It is well established that telomere shortening occurs with cellular division, and prolonged culture of ADSCs has been linked to a decline in differentiation potential and replicative aging, ultimately leading to senescence (Bieback et al., 2012; Bonab et al., 2006). In the absence of specific differentiation cues in standard culture media, ADSCs may retain their MSC-like state rather than progressing toward non-native lineages. The current findings suggest that ADSCs maintained in standard culture conditions may be more inclined toward chondrogenic differentiation, as evidenced by the upregulation of genes associated with cartilage development and chondrocyte differentiation in the osteoblast control group. This propensity could be influenced by the source of the cells, their cellular memory, and the methylation status of key transcription factors that govern cell fate decisions (Xu et al., 2017; Jääger et al., 2012).

Although ADSCs have the potential to differentiate into neurogenic lineages, this differentiation is non-canonical and typically inefficient (Locke et al., 2011). However, both neuronal and glial markers, such as NSE, NeuN, or vimentin, were identified even in undifferentiated ADSCs and other MSCs (Safford and Rice, 2005). Human MSCs after osteogenic and adipogenic differentiation also expressed neuronal markers, namely, NeuN and βIII-tubulin (Foudah et al., 2013). In contrast, the present study revealed a decrease in the expression of genes related to neurogenesis and gliogenesis following osteogenic differentiation of canine ADSCs. Similarly, Shai et al. (2019) reported the downregulation of nervous system development-related genes and others associated with neurogenesis. A recent study suggests that osteogenic and neurogenic differentiation may be inversely regulated in ADSCs via the MAPK-mediated signaling pathway, with epiregulin promoting osteogenic differentiation while inhibiting neurogenic potential (Cao et al., 2020).

Consistently with these findings, the pathway enrichment analysis revealed over-representation of “axon guidance” pathway in OC *versus* EC comparison and under-representation in both OT *versus* EC and OT *versus* OC comparisons. Axon guidance factors comprise versatile proteins involved in regulation of both physiological and pathological processes, which are particularly active during nerve growth and regeneration. In addition, these proteins were demonstrated to be involved in osteogenic differentiation and bone formation, either by inhibitor of promotion of this processes (Liu et al., 2022). On the contrary to current results, “axon guidance” pathway was shown to be positively enriched in osteogenically differentiated BM-MSCs (Wan et al., 2021), indicating the need to perform further research regarding its role in MSCs osteogenic differentiation.

In osteo-induced ADSCs, a significant downregulation of genes associated with various morphogenic processes, such as “gland morphogenesis,” “animal organ morphogenesis,” “salivary gland morphogenesis,” “prostate gland morphogenesis,” and “tissue morphogenesis,” was observed compared to the early control group (EC). This suggests that ADSCs without differentiation cues remain in a more multipotent state, maintaining higher expression of genes involved in general morphogenesis (Wagner et al., 2005). In contrast, osteo-induced ADSCs become committed to the osteogenic lineage, leading to the upregulation of osteogenic differentiation-related genes and the corresponding downregulation of genes associated with other morphogenic pathways.

## Conclusion

In summary, the current results provide valuable insights into the transcriptomic changes associated with key biological processes in canine ADSCs following osteogenic differentiation. The upregulation of genes involved in apoptosis regulation suggests a balance between cell survival and death, ensuring the quality of differentiating osteoblasts and the removal of damaged cells, which is critical for proper tissue development. Additionally, the upregulation of genes related to circulatory system development reinforces the role of differentiating ADSCs in supporting blood vessel formation and remodeling—an essential aspect of bone tissue growth and repair, with potential applications in bone tissue engineering. Furthermore, the observed downregulation of genes involved in neurogenesis and gliogenesis suggests a reciprocal regulation of these processes during osteogenic differentiation.

It is important to note that 2D *in vitro* cultures do not fully replicate the complexity of *in vivo* conditions, where cytokines, growth factors, and the cellular microenvironment play critical roles in determining the fate of ADSCs and guiding their differentiation. While transcriptomic analysis provides valuable insights into gene expression, it has limitations in capturing post-transcriptional modifications and protein activity. Therefore, complementary proteomic studies are required to fully understand the functional protein dynamics and their regulatory roles in osteogenic differentiation of ADSCs. Moreover, further functional and *in vivo* studies, as well as a deeper understanding of the signaling networks and transcription factors involved in ADSC osteogenic differentiation, are necessary to develop safe and effective stem cell therapies. However, *in vitro* models are invaluable for identifying key biological processes in osteo-induced ADSCs and highlighting potential targets for enhancing and optimizing differentiation. Extending this research to other MSC types will undoubtedly advance stem cell therapies and tissue engineering efforts.

## Data Availability

The datasets presented in this study can be found in online repositories. The names of the repository/repositories and accession number(s) can be found below: https://www.ncbi.nlm.nih.gov/geo/, GSE280031.

## Supplementary material

SUPPLEMENTARY TABLE S1 Complete list of DEGs analysed in this manuscript. The list includes gene names, logFC, *p*-values and adjusted *p*-values, as well as the information which comparison the logFC relates to (EC-early control, OC-late control, OT-differentiated osteoblasts).

SUPPLEMENTARY TABLE S2 Complete results of GO and KEGG pathway enrichment performed in this study. The results include GO numbers and names, enrichment gene ratios, *p*-values, q-values, information if the enrichment was positive or negative, as well as the information which comparison the enrichment relates to (EC-early control, OC-late control, OT-differentiated osteoblasts).

## References

[B1] Andrzejewska A. Lukomska B. Janowski M. (2019). Concise review: mesenchymal stem cells: from roots to boost. Stem Cells 37, 855–864. 10.1002/stem.3016 30977255 PMC6658105

[B2] Armesilla-Diaz A. Elvira G. Silva A. (2009). P53 regulates the proliferation, differentiation and spontaneous transformation of mesenchymal stem cells. Exp. Cell Res. 315, 3598–3610. 10.1016/J.YEXCR.2009.08.004 19686735

[B3] Bajek A. Gurtowska N. Olkowska J. Maj M. Kaźmierski Ł. Bodnar M. (2017). Does the harvesting technique affect the properties of adipose-derived stem cells? the comparative biological characterization. J. Cell Biochem. 118, 1097–1107. 10.1002/jcb.25724 27608167

[B4] Bieback K. Hecker A. Schlechter T. Hofmann I. Brousos N. Redmer T. (2012). Replicative aging and differentiation potential of human adipose tissue-derived mesenchymal stromal cells expanded in pooled human or fetal bovine serum. Cytotherapy 14, 570–583. 10.3109/14653249.2011.652809 22300364

[B5] Bonab M. M. Alimoghaddam K. Talebian F. Ghaffari S. H. Ghavamzadeh A. Nikbin B. (2006). Aging of mesenchymal stem cell *in vitro* . BMC Cell Biol. 7, 14. 10.1186/1471-2121-7-14 16529651 PMC1435883

[B6] Brennan M. A. Renaud A. Guilloton F. Mebarki M. Trichet V. Sensebé L. (2017). Inferior *in vivo* osteogenesis and superior angiogeneis of human adipose tissue: a comparison with bone marrow‐derived stromal stem cells cultured in xeno‐free conditions. Stem Cells Transl. Med. 6, 2160–2172. 10.1002/SCTM.17-0133 29052365 PMC5702520

[B7] Bunnell B. A. (2021). Adipose tissue-derived mesenchymal stem cells. Cells 10, 3433. 10.3390/CELLS10123433 34943941 PMC8700397

[B8] Cao Y. Shi R. Yang H. Zhang J. Ge L. Gao R. (2020). Epiregulin promotes osteogenic differentiation and inhibits neurogenic trans-differentiation of adipose-derived mesenchymal stem cells via MAPKs pathway. Cell Biol. Int. 44, 1046–1058. 10.1002/CBIN.11305 31930610

[B9] Cao Y. Sun Z. Liao L. Meng Y. Han Q. Zhao R. C. (2005). Human adipose tissue-derived stem cells differentiate into endothelial cells *in vitro* and improve postnatal neovascularization *in vivo* . Biochem. Biophys. Res. Commun. 332, 370–379. 10.1016/J.BBRC.2005.04.135 15896706

[B10] Ceccarelli S. Pontecorvi P. Anastasiadou E. Napoli C. Marchese C. (2020). Immunomodulatory effect of adipose-derived stem cells: the cutting edge of clinical application. Front. Cell Dev. Biol. 8, 531513. 10.3389/fcell.2020.00236 PMC7180192 32363193

[B11] de Bakker E. Van Ryssen B. De Schauwer C. Meyer E. (2013). Canine mesenchymal stem cells: state of the art, perspectives as therapy for dogs and as a model for man. Vet. Q. 33, 225–233. 10.1080/01652176.2013.873963 24404887

[B12] Durandt C. Dessels C. da Silva C. Murdoch C. Pepper M. S. (2019). The effect of early rounds of *ex vivo* expansion and cryopreservation on the adipogenic differentiation capacity of adipose-derived stromal/stem cells. Sci. Rep. 9, 15943. 10.1038/s41598-019-52086-9 31685852 PMC6828715

[B13] Feisst V. Meidinger S. Locke M. B. (2015). From bench to bedside: use of human adipose-derived stem cells. Stem Cells Cloning 8, 149–162. 10.2147/SCCAA.S64373 26586955 PMC4636091

[B14] Foudah D. Redondo J. Caldara C. Carini F. Tredici G. Miloso M. (2013). Human mesenchymal stem cells express neuronal markers after osteogenic and adipogenic differentiation. Cell Mol. Biol. Lett. 18, 163–186. 10.2478/s11658-013-0083-2 23430457 PMC6275956

[B15] Francis S. L. Duchi S. Onofrillo C. Di Bella C. Choong P. F. M. (2018). Adipose-derived mesenchymal stem cells in the use of cartilage tissue engineering: the need for a rapid isolation procedure. Stem Cells Int. 2018, 8947548. 10.1155/2018/8947548 29765427 PMC5903192

[B16] Fujita T. Azuma Y. Fukuyama R. Hattori Y. Yoshida C. Koida M. (2004). Runx2 induces osteoblast and chondrocyte differentiation and enhances their migration by coupling with PI3K-akt signaling. J. Cell Biol. 166, 85–95. 10.1083/JCB.200401138 15226309 PMC2172136

[B17] Gao S. Chen B. Zhu Z. Du C. Zou J. Yang Y. (2023). PI3K-Akt signaling regulates BMP2-induced osteogenic differentiation of mesenchymal stem cells (MSCs): a transcriptomic landscape analysis. Stem Cell Res. 66, 103010. 10.1016/J.SCR.2022.103010 36580886

[B18] Gavin K. M. Bessesen D. H. (2020). Sex differences in adipose tissue function. Endocrinol. Metab. Clin. North Am. 49, 215–228. 10.1016/J.ECL.2020.02.008 32418585 PMC7921847

[B19] Gentleman R. C. Carey V. J. Bates D. M. Bolstad B. Dettling M. Dudoit S. (2004). Bioconductor: open software development for computational biology and bioinformatics. Genome Biol. 5 (5), R80–R16. 10.1186/GB-2004-5-10-R80 15461798 PMC545600

[B20] Gibler P. Gimble J. Hamel K. Rogers E. Henderson M. Wu X. (2021). Human adipose-derived stromal/stem cell culture and analysis methods for adipose tissue modeling *in vitro*: a systematic review. Cells 10, 1378. 10.3390/CELLS10061378 34204869 PMC8227575

[B21] Hernández Borrero L. J. El-Deiry W. S. (2021). Tumor suppressor P53: biology, signaling pathways, and therapeutic targeting. Biochim. Biophys. Acta Rev. Cancer 1876, 188556. 10.1016/J.BBCAN.2021.188556 33932560 PMC8730328

[B22] Hoffman A. M. Dow S. W. (2016). Concise review: stem cell trials using companion animal disease models. Stem Cells 34, 1709–1729. 10.1002/STEM.2377 27066769

[B23] Jääger K. Islam S. Zajac P. Linnarsson S. Neuman T. (2012). RNA-seq analysis reveals different dynamics of differentiation of human dermis- and adipose-derived stromal stem cells. PLoS One 7, e38833. 10.1371/JOURNAL.PONE.0038833 22723894 PMC3378616

[B24] Jain A. K. Barton M. C. (2018). P53: emerging roles in stem cells, development and beyond. Dev. Camb. 145, 145. 10.1242/dev.158360 29654218

[B25] Jankowski M. Dompe C. Sibiak R. Wąsiatycz G. Mozdziak P. Jaśkowski J. M. (2020). *In vitro* cultures of adipose-derived stem cells: an overview of methods, molecular analyses, and clinical applications. Cells 9, 1783. 10.3390/cells9081783 32726947 PMC7463427

[B26] Jankowski M. Kaczmarek M. Wąsiatycz G. Dompe C. Mozdziak P. Jaśkowski J. M. (2021). Expression profile of new marker genes involved in differentiation of canine adipose-derived stem cells into osteoblasts. Int. J. Mol. Sci. 22, 6663. 10.3390/IJMS22136663 34206369 PMC8269079

[B27] Jankowski M. Kaczmarek M. Wąsiatycz G. Konwerska A. Dompe C. Bukowska D. (2022). Expression profile of new gene markers involved in differentiation of canine adipose-derived stem cells into chondrocytes. Genes (Basel) 13, 1664. 10.3390/GENES13091664 36140831 PMC9498306

[B28] Kanczler J. M. Oreffo R. O. C. (2008). Osteogenesis and angiogenesis: the potential for engineering bone. Eur. Cell Mater 15, 100–114. 10.22203/ECM.V015A08 18454418

[B29] Lee H.-J. Park B.-J. Jeon R.-H. Jang S.-J. Son Y.-B. Lee S.-L. (2019). Alteration of apoptosis during differentiation in human dental pulp-derived mesenchymal stem cell. J. Animal Reproduction Biotechnol. 34, 2–9. 10.12750/JARB.34.1.2

[B30] Liu J. Yao Y. Huang J. Sun H. Pu Y. Tian M. (2022). Comprehensive analysis of LncRNA-MiRNA-MRNA networks during osteogenic differentiation of bone marrow mesenchymal stem cells. BMC Genomics 23, 425. 10.1186/S12864-022-08646-X 35672672 PMC9172120

[B31] Locke M. Feisst V. Dunbar P. R. (2011). Concise review: human adipose-derived stem cells: separating promise from clinical need. Stem Cells 29, 404–411. 10.1002/STEM.593 21425404

[B32] Lo Furno D. Graziano A. C. E. Caggia S. Perrotta R. E. Tarico M. S. Giuffrida R. (2013). Decrease of apoptosis markers during adipogenic differentiation of mesenchymal stem cells from human adipose tissue. Apoptosis 18, 578–588. 10.1007/S10495-013-0830-X 23479126

[B33] Love M. I. Huber W. Anders S. (2014). Moderated estimation of fold change and dispersion for RNA-seq data with DESeq2. Genome Biol. 15, 550–621. 10.1186/s13059-014-0550-8 25516281 PMC4302049

[B34] Mende W. Götzl R. Kubo Y. Pufe T. Ruhl T. Beier J. P. (2021). The role of adipose stem cells in bone regeneration and bone tissue engineering. Cells 10, 975. 10.3390/CELLS10050975 33919377 PMC8143357

[B35] Merlo B. Iacono E. (2023). Beyond canine adipose tissue-derived mesenchymal stem/stromal cells transplantation: an update on their secretome characterization and applications. Anim. (Basel) 13, 3571. 10.3390/ANI13223571 PMC10668816 38003188

[B36] Neri S. Bourin P. Peyrafitte J. A. Cattini L. Facchini A. Mariani E. (2013). Human adipose stromal cells (ASC) for the regeneration of injured cartilage display genetic stability after *in vitro* culture expansion. PLoS One 8, e77895. 10.1371/journal.pone.0077895 24205017 PMC3810264

[B37] Oliver L. Hue E. Rossignol J. Bougras G. Hulin P. Naveilhan P. (2011). Distinct roles of Bcl-2 and Bcl-Xl in the apoptosis of human bone marrow mesenchymal stem cells during differentiation. PLoS One 6, e19820. 10.1371/JOURNAL.PONE.0019820 21589877 PMC3093403

[B38] Oliver L. Hue E. Séry Q. Lafargue A. Pecqueur C. Paris F. (2013). Differentiation-related response to DNA breaks in human mesenchymal stem cells. Stem Cells 31, 800–807. 10.1002/STEM.1336 23341263

[B39] Ong W. K. Chakraborty S. Sugii S. (2021). Adipose tissue: understanding the heterogeneity of stem cells for regenerative medicine. Biomolecules 11, 918. 10.3390/biom11070918 34206204 PMC8301750

[B40] Palumbo P. Lombardi F. Siragusa G. Cifone M. G. Cinque B. Giuliani M. (2018). Methods of isolation, characterization and expansion of human adipose-derived stem cells (ASCs): an overview. Int. J. Mol. Sci. 19, 1897. 10.3390/ijms19071897 29958391 PMC6073397

[B41] Reya T. Morrison S. J. Clarke M. F. Weissman I. L. (2001). Stem cells, cancer, and cancer stem cells. Nature 414, 105–111. 10.1038/35102167 11689955

[B42] Robert A. W. Angulski A. B. B. Spangenberg L. Shigunov P. Pereira I. T. Bettes P. S. L. (2018). Gene expression analysis of human adipose tissue-derived stem cells during the initial steps of *in vitro* osteogenesis. Sci. Rep. 8 (8), 4739–4811. 10.1038/s41598-018-22991-6 29549281 PMC5856793

[B43] Romero A. Barrachina L. Ranera B. Remacha A. R. Moreno B. de Blas I. (2017). Comparison of autologous bone marrow and adipose tissue derived mesenchymal stem cells, and platelet rich plasma, for treating surgically induced lesions of the equine superficial digital flexor tendon. Veterinary J. 224, 76–84. 10.1016/j.tvjl.2017.04.005 28697880

[B44] Safford K. Rice H. (2005). Stem cell therapy for neurologic disorders: therapeutic potential of adipose-derived stem cells. Curr. Drug Targets 6, 57–62. 10.2174/1389450053345028 15720213

[B45] Shaik S. Martin E. C. Hayes D. J. Gimble J. M. Devireddy R. V. (2019). Transcriptomic profiling of adipose derived stem cells undergoing osteogenesis by RNA-seq. Sci. Rep. 9, 11800. 10.1038/s41598-019-48089-1 31409848 PMC6692320

[B46] Stefańska K. Nemcova L. Blatkiewicz M. Pieńkowski W. Ruciński M. Zabel M. (2023). Apoptosis related human wharton’s jelly-derived stem cells differentiation into osteoblasts, chondrocytes, adipocytes and neural-like cells—complete transcriptomic assays. Int. J. Mol. Sci. 24, 10023. 10.3390/ijms241210023 37373173 PMC10297881

[B47] Steiner B. M. Berry D. C. (2022). The regulation of adipose tissue health by estrogens. Front. Endocrinol. (Lausanne) 13, 889923. 10.3389/FENDO.2022.889923 35721736 PMC9204494

[B48] Tataria M. Quarto N. Longaker M. T. Sylvester K. G. (2006). Absence of the P53 tumor suppressor gene promotes osteogenesis in mesenchymal stem cells. J. Pediatr. Surg. 41, 624–632. 10.1016/J.JPEDSURG.2005.12.001 16567167

[B49] Wagner W. Wein F. Seckinger A. Frankhauser M. Wirkner U. Krause U. (2005). Comparative characteristics of mesenchymal stem cells from human bone marrow, adipose tissue, and umbilical cord blood. Exp. Hematol. 33, 1402–1416. 10.1016/J.EXPHEM.2005.07.003 16263424

[B50] Wan Q. Q. Qin W. P. Ma Y. X. Shen M. J. Li J. Zhang Z. B. (2021). Crosstalk between bone and nerves within bone. Adv. Sci. 8, 2003390. 10.1002/ADVS.202003390 PMC8025013 33854888

[B51] Wang Z. Gerstein M. Snyder M. (2009). RNA-seq: a revolutionary tool for transcriptomics. Nat. Rev. Genet. 10, 57–63. 10.1038/NRG2484 19015660 PMC2949280

[B52] Wu T. Hu E. Xu S. Chen M. Guo P. Dai Z. (2021). ClusterProfiler 4.0: a universal enrichment tool for interpreting omics data. Innov. Camb. (Mass.) 2, 100141. 10.1016/J.XINN.2021.100141 PMC8454663 34557778

[B53] Xu L. Liu Y. Sun Y. Wang B. Xiong Y. Lin W. (2017). Tissue source determines the differentiation potentials of mesenchymal stem cells: a comparative study of human mesenchymal stem cells from bone marrow and adipose tissue. Stem Cell Res. Ther. 8, 275. 10.1186/S13287-017-0716-X 29208029 PMC5718061

[B54] Youle R. J. Strasser A. (2008). The BCL-2 protein family: opposing activities that mediate cell death. Nat. Rev. Mol. Cell Biol. 9, 47–59. 10.1038/NRM2308 18097445

